# Enantioselective One-Pot Synthesis of α-Amino Esters by a Phosphine-Catalyzed [3+2]-Cycloaddition Reaction

**DOI:** 10.1002/chem.201103502

**Published:** 2011-12-07

**Authors:** Marianne Steurer, Kim L Jensen, Dennis Worgull, Karl Anker Jørgensen

**Affiliations:** [a] Center for Catalysis, Department of Chemistry, Aarhus University8000 Aarhus C (Denmark)

**Keywords:** amino esters, asymmetric synthesis, cycloaddition, organocatalysis, phosphines

Phosphine-catalyzed [3+2]-cycloaddition reactions of electron-deficient allenes and alkynes with α,β-unsaturated carbonyl compounds can give access to important highly functionalized cyclopentenes.^1^ The seminal example of such a transformation was first reported by Lu et al. in 1995,^2^ and its asymmetric version in 1997 by the group of Zhang.^3^ However, it took another ten years before the potential of this annulation strategy using chiral phosphine catalysts was studied more intensively.^4^ The last decade has witnessed considerable progress in the development of suitable new chiral phosphine catalysts and their application in asymmetric [3+2]-cycloaddition reactions.^5^

**Scheme 1:**
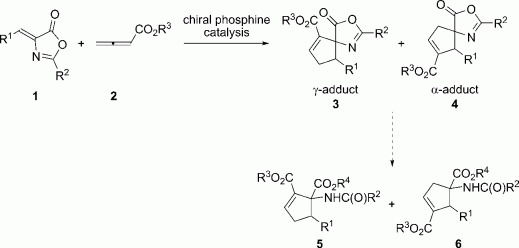
Synthesis of optically active α-amino esters from allenes and olefinic azlactones.

Recently, readily available olefinic azlactones have emerged as amino acid precursors in organocatalysis.^6^ Therefore, we decided to develop a novel asymmetric phosphine-catalyzed [3+2]-cycloaddition reaction of olefinic azlactones with allenes for the synthesis of highly functionalized chiral amino esters. The cycloaddition between olefinic azlactones **1** and allenes **2** using a chiral phosphine catalyst would thereby give access to enantioenriched cyclopentenyl derivatives **3** and **4** (Scheme 1).

To the best of our knowledge, the optically active cyclopentenyl derivatives **3** and **4** have until now only been synthesized efficiently in a racemic manner.^7^, ^8^ Herein, we report the results of our studies on the enantioselective one-pot synthesis of cyclic α-amino esters by a phosphine-catalyzed [3+2]-cycloaddition reaction of azlactones with allenes. In general, two pathways are possible for this reaction, affording regioisomers resulting from α- or γ-addition. Since β-substituted exocyclic enones preferentially undergo γ-addition,^3^, 5m,o we assumed that the γ-adduct would be favored, giving access to aspartic acid derivatives **5** after a ring-opening reaction of the azlactone moiety.

**Scheme 2:**
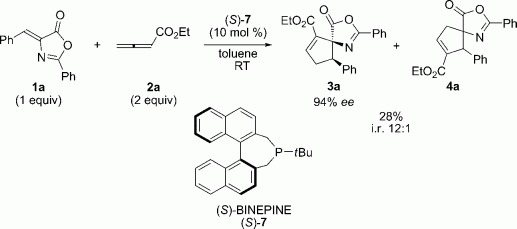
[3+2]-Cycloaddition reaction of (*Z*)-olefinic azlactone **1 a** with ethyl buta-2,3-dienoate **2 a** catalyzed by (*S*)-BINEPINE.

At the outset, a number of chiral phosphine catalysts were evaluated for the [3+2]-cycloaddition reaction of (*Z*)-olefinic azlactone **1 a** with ethyl buta-2,3-dienoate **2 a** (see the Supporting Information). Among the catalysts tested, (*S*)-BINEPINE^9^ afforded the highest regio- and enantioselectivities. As anticipated, it was found that the γ-addition was favored, providing a 12:1 regioisomeric ratio (i.r.) of products **3 a** and **4 a** with an enantiomeric excess of 94 % *ee* of **3 a** (Scheme 2).^10^ Additional screening of solvents and concentrations showed that the best results were obtained in toluene and a concentration of 0.1 m provided a better solubility of the azlactone. It should be noted that no difference in the reaction outcome was observed when the reaction was performed under air or inert conditions, which enhances the practicability and usefulness of this phosphine-catalyzed cycloaddition. A screening of different allenes showed that the ethyl ester **2 a** in combination with an azlactone containing a phenyl group at the C2 position (R^2^=Ph, see Scheme 1), provided the highest, yet disappointing, conversion and selectivities. The cycloaddition with Me-substituted azlactone (R^1^=Ph, R^2^=Me) resulted in inferior results.

**Scheme 3:**
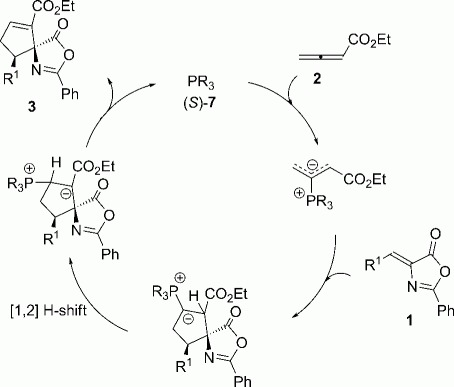
Proposed mechanism for the phosphine-catalyzed [3+2]-cycloaddition reaction.

Since only partial conversion of the azlactone **1 a**, and therefore a low combined yield of products **3 a** and **4 a**, was observed in the cycloaddition (28 %, Table 1, entry 1), an optimization of the reaction conditions was performed. An increase of the catalyst loading to 20 mol % resulted in a significantly higher yield (47 %), though still remaining unsatisfactory low (Table 1, entry 2). Monitoring the reaction showed that the degree of conversion of the azlactone to the products **3 a** and **4 a** did not increase after three hours (perhaps due to catalyst inhibition). Mechanistic studies on phosphine-catalyzed [3+2]-cycloaddition reactions by Yu et al. suggest the necessity of water to induce the 1,2-proton transfer to release the catalyst from the intermediate ylide species.^2^, ^11^ Scheme 3 outlines the mechanistic proposal for the catalytic cycle. Therefore, several additives such as water, alcohols, acids, as well as buffered systems, to promote this proton shift were tested. However, none of these additives improved the conversion of the transformation (results not shown).

**1:** Optimization of reaction conditions.^[a]^
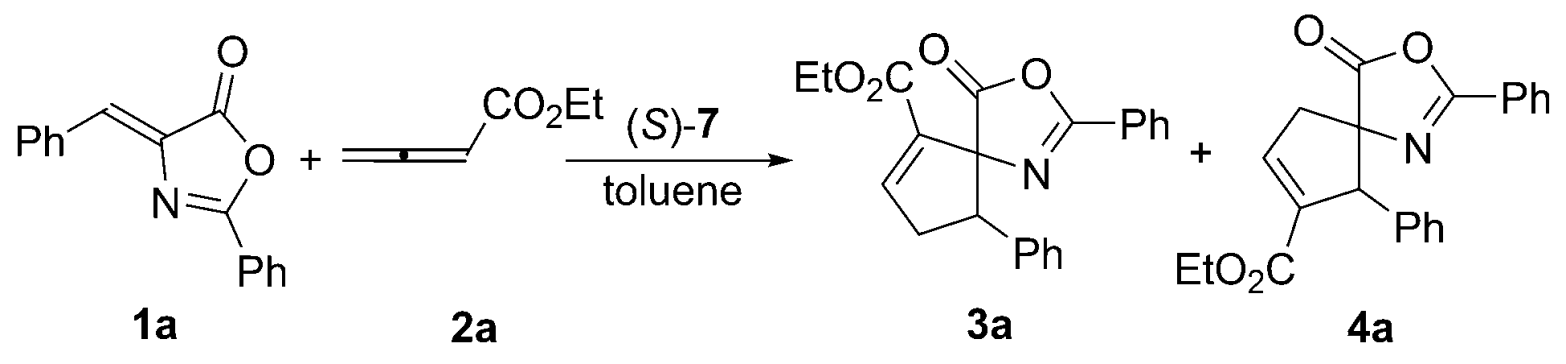

Entry	(*S*)-**7** [mol %]	Time/Temp [h/°C]	Yield [%]^[b]^	i.r. [**3a**:**4a**]^[c]^	*ee* [%]^[d]^
1^[e]^	10	22/RT	28	12:1	94
2^[e]^	20	17/RT	47	ca. 10:1	94
3	20	2/110	72	4:1	85
4	20	21/60	55	7:1	92
5	20	21/80	66	5:1	89
6	10	22/80	25	5:1	n.d.^[f]^
7	20	3/RT then 21/110	83	4:1	91
**8**	**20**	3/RT then 21/80	**77**	**5:1**	**93**

[a] **1 a** (0.1 mmol), **2 a** (0.15 mmol), and (*S*)-**7** in toluene (1.0 mL). [b] Isolated yield of **3** and **4** after FC. [c] i.r.=regioisomeric ratio; determined by ^1^H NMR spectroscopy of the crude reaction mixture. [d] Determined on **5 a** by HPLC analysis using a chiral stationary phase. [e] Performed under argon; with **2 a** (0.2 mmol) and toluene (0.5 mL). [f] n.d.=not determined.

Finally, the effect of the reaction temperature was investigated. Preliminary experiments at lower temperatures showed that at 5 °C almost the same conversion was obtained as at room temperature, whereas the reaction was very slow at −20 °C. Refluxing in toluene for 2 h led to almost complete consumption of **1 a**, providing a significantly higher combined yield of 72 % of products **3 a** and **4 a** (47 % obtained at room temperature, compare Table 1, entries 2 and 3). While the regioselectivity dropped from 10:1 to 4:1, the enantioselectivity was, to our surprise, only reduced to 85 % *ee*. A screening of different reaction temperatures showed that by performing the reaction at room temperature followed by heating to 80 °C gave the best combined results with respect to yield, regioselectivity, and enantioselectivity (Table 1, entry 8). An attempt to reduce the catalyst loading to 10 mol % resulted in low conversion of **1 a** (Table 1, entry 6), and the following experiments were therefore performed with 20 mol % ofBINEPINE (*S*)-**7**.

**Scheme 4:**
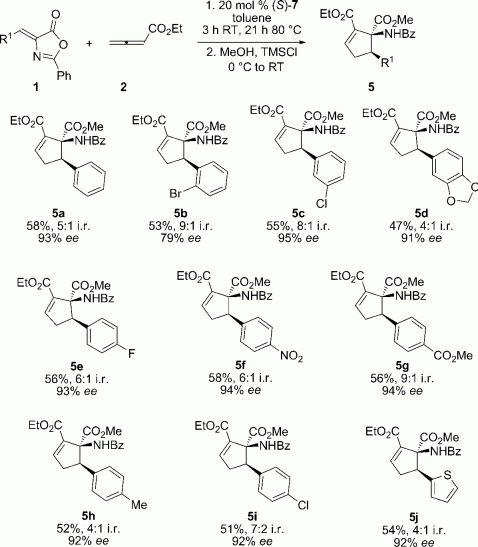
Scope of the one-pot sequence to optically active amino esters. Yields are given for the major isomer (i.r.=regioisomeric ratio of **3**:**4** determined after the first step).

Based on the optimization of the first reaction step, we next envisioned developing a one-pot [3+2]-cycloaddition/methanolysis procedure that provides direct access to protected amino acid derivatives. Gratifyingly, treating the crude reaction mixture with MeOH/TMSCl resulted in the opening of azlactones **3** and **4** to give the products **5** and **6**, respectively. For the reaction of (*Z*)-olefinic azlactone **1 a** with ethyl buta-2,3-dienoate **2 a**, pure major isomer **5 a** was isolated in a good overall yield (58 %, Scheme 4) in conjunction with a high enantioselectivity (93 % *ee*). The scope of the reaction was therefore studied, using this developed one-pot sequence (Scheme 4). All optically active amino acid derivatives were isolated as single regioisomers **5**, and the given regioisomeric ratios were determined after the first step. Different β-aryl-substituted azlactones were evaluated, including a variation of substituents containing electron-donating, as well as electron-withdrawing functionalities on the aryl moiety (products **5 b**–**i**). Furthermore, a heteroaromatic azlactone was successfully employed, thereby broadening the scope of the reaction (product **5 j**). In general, very high enantioselectivities (>90 % *ee*) were obtained with regioselectivities between 7:2 and 9:1. The yields of this one-pot sequence to amino esters **5** were comparable for all substrates. Only in the case of *ortho*-bromo aryl-substituted azlactone **1 b**, the enantioselectivity dropped to 79 % *ee*, whilst the yield and the regioselectivity remained in the same range as shown for the other substrates.

This new one-pot reaction can be classified as a TypeA-2-2C2X^12^ sequence and provides an easy approach to enantioenriched amino acid derivatives containing a quarternary stereocenter.

The absolute configuration of the major isomer **3 a** was determined to be (5*S*,9*R*) by single-crystal X-ray analysis (see Figure 1).^13^ The stereochemistry of amino ester **5 a** was assigned according to **3 a** to be (1*S*,5*R*) and assuming a common reaction pathway, the configuration of the other derivatives **5** was assigned by analogy.

**1:**
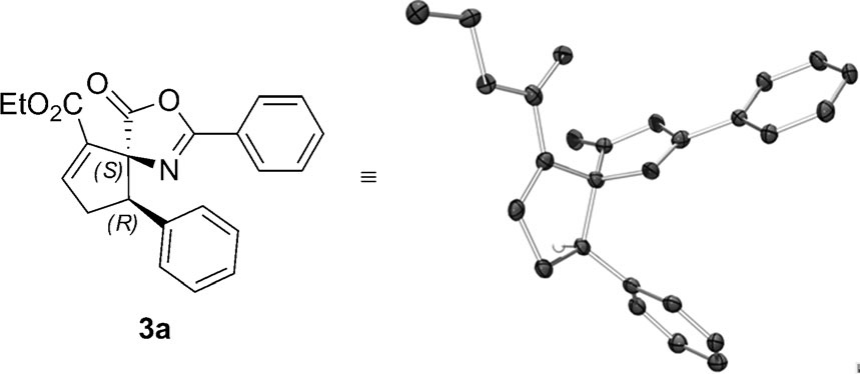
X-ray structure of the major isomer a

**Scheme 5:**
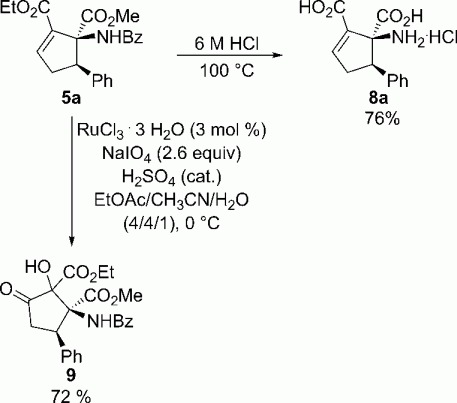
Hydrolysis and oxidation of amino ester **5 a**.

The obtained optically active amino acid derivatives can undergo a number of important transformations. The amino acids **8** are readily available by hydrolysis of amino esters **5** as shown for derivative **8 a**, which was isolated as its HCl salt in 76 % yield (Scheme 5).^8^ Functionalization of the α,β-unsaturated ester moiety could be achieved by a Ru-catalyzed ketohydroxylation of product **5 a**, and resulted in α-hydroxy-β-ketoester **9** in 72 % yield.^14^

In conclusion, we have developed a TypeA-2-2C2X^12^ one-pot synthesis of enantioenriched amino acid derivatives containing a quarternary stereocenter. The sequence relies on an asymmetric phosphine-catalyzed [3+2]-cycloaddition reaction between ethyl buta-2,3-dienoate and (*Z*)-olefinic azlactones, followed by an in situ ring opening of the azlactone moiety. The products were generally obtained in good overall yields and high enantioselectivities. Transformations of the obtained amino esters to the respective amino acid and to an α-hydroxy-β-ketoester have successfully been demonstrated, giving access to further functionalizable compounds.
